# Advances in Radiological Imaging Modalities and Their Expanding Role in the Early Diagnosis, Monitoring, and Prognosis of Internal Medicine Disorders: A Comprehensive Review

**DOI:** 10.7759/cureus.99513

**Published:** 2025-12-18

**Authors:** Rishitha Rao Ketineni, Bhanupriya Singh, Achsah Raj Chandralekha, Indurani M S, Kanak Soni, Niraj Lodha

**Affiliations:** 1 Department of Medicine, Jagadguru Sri Shivarathreeshwara Medical College, Mysore, IND; 2 Department of Radio-Diagnosis, Sanjay Gandhi Postgraduate Institute of Medical Sciences, Lucknow, IND; 3 Department of Medicine, Christian Medical College and Hospital, Ludhiana, IND; 4 Department of Radio-Diagnosis, SRM Medical College Hospital and Research Centre, SRM Institute of Science and Technology, Kattankulathur, IND; 5 Department of Naturopathy and Yogic Sciences, University of Patanjali, Haridwar, IND; 6 Department of Medicine, Lodha Hospital and Research Centre, Pali, IND

**Keywords:** artificial intelligence, early diagnosis, multimodal imaging, personalized medicine, radiological imaging

## Abstract

This review explores the growing role of advanced radiological imaging in internal medicine, focusing on its applications in prognosis prediction, disease monitoring, and early diagnosis. It highlights how developments in computed tomography (CT), magnetic resonance imaging (MRI), positron emission tomography (PET), ultrasound, and the integration of artificial intelligence (AI) are reshaping clinical decision-making in fields such as neurology, cardiology, and oncology. Although progress has been substantial, widespread adoption is still limited by high costs, unequal access, and the absence of consistent protocols. The use of AI in combination with radiomics, the quantitative study of medical images, has enhanced diagnostic accuracy and expanded opportunities for outcome prediction and treatment planning. However, challenges remain, including inconsistencies in data quality, regulatory barriers, and the pressing need for validation through large-scale, multicenter studies. Hybrid technologies such as PET/MRI, which combine functional and anatomical imaging, hold particular promise for improving diagnostic precision in neurology and oncology. Together, these innovations illustrate the transformative potential of modern imaging to enable earlier interventions and support more personalized care strategies. The review emphasizes the need for validation, standardized frameworks, and international collaboration to overcome current limitations. Addressing these concerns will broaden the accessibility of these tools, fostering more equitable healthcare and improved outcomes worldwide. Finally, it provides practical guidance for clinicians, researchers, and policymakers who must adapt to the rapidly advancing landscape of radiological imaging.

## Introduction and background

In internal medicine, radiological imaging has reshaped the way physicians detect, track, and manage disease. What started with simple X-rays has evolved into advanced tools, including computed tomography (CT), magnetic resonance imaging (MRI), and positron emission tomography (PET), which are now integral to everyday clinical decision-making [1]. Because internal medicine encompasses a broad spectrum of conditions, many with subtle or non-specific early signs, physical examination on its own often cannot provide reliable detection [2]. Imaging can identify disease before clinical symptoms emerge, supporting earlier treatment and improving patient outcomes [3]. Advances in imaging now extend beyond conventional anatomic visualization to include techniques such as radiomics, hybrid PET/MRI systems, and ultrasound elastography. Recent reviews emphasise that modern imaging increasingly integrates precision medicine principles, combining multimodal data to improve diagnostic performance [4]. This review examines developments across CT, MRI, PET, ultrasound-based imaging, and computational techniques across oncology, cardiology, neurology, and metabolic disease. Modern innovations have extended imaging beyond structural assessment to include molecular, metabolic, and functional information [5]. Hybrid imaging modalities such as PET/MRI and PET/CT continue to expand clinical utility and diagnostic accuracy [6]. Developments in CT, such as dual-energy acquisition and iterative reconstruction, allow more precise tissue characterization while lowering radiation exposure [6]. Recent evidence confirms that these CT innovations significantly enhance tissue differentiation and reduce noise [4]. In neurology, diffusion-weighted imaging has become essential in acute stroke, where it can reveal ischemic changes within minutes. In cardiology, cardiac MRI provides detailed insights into myocardial viability and fibrosis, guiding advanced treatment strategies [7].

PET combines metabolic and structural information, long indispensable in oncology, and increasingly valuable in cardiovascular and inflammatory diseases, particularly with hybrid modalities such as PET/CT and PET/MRI [8]. Contemporary literature highlights continual improvements in PET biomarkers and molecular imaging for cancer and inflammatory disease detection [5]. Elastography has advanced imaging by enhancing contrast in vascular and abdominal studies while making ultrasound widely accessible for liver fibrosis staging [9]. Recent reports further highlight ultrasound’s critical role in resource-limited regions and its growing importance in global health imaging [6]. Although progress has been substantial, many publications examine single modalities, with fewer offering an integrated overview across internal medicine. Calls for cross-disciplinary integration, combining radiology, computational analytics, and precision medicine strategies, have become increasingly prominent in recent reviews [10].

Imaging is also central in monitoring and prognosis. PET and functional MRI biomarkers of tumor metabolism and vascularity are widely used in oncology to monitor therapy response and modify treatment plans before progression [11]. Cardiac MRI provides prognostic metrics such as late gadolinium enhancement and ejection fraction that are stronger predictors of outcomes than echocardiography. MRI enterography offers non-radiation monitoring for inflammatory bowel disease [12]. Recent studies highlight the expanding role of quantitative imaging biomarkers in long-term surveillance of cardiac and neurodegenerative diseases [13]. Along with hardware advances, radiology is being transformed by computational techniques [14]. Machine learning and artificial intelligence (AI) are increasingly applied to image acquisition, reconstruction, and interpretation to improve diagnostic accuracy and reveal patterns not readily detected by human observers [15]. Recent work also emphasises reproducibility challenges in radiomics, including scanner variability and protocol inconsistency [16]. Without improvements in data standardization, multicenter validation, and regulatory transparency, computational imaging will remain largely experimental [17]. Recent reviews confirm the need for robust validation frameworks and clear regulatory pathways for AI in imaging [18].

**Figure 1:**
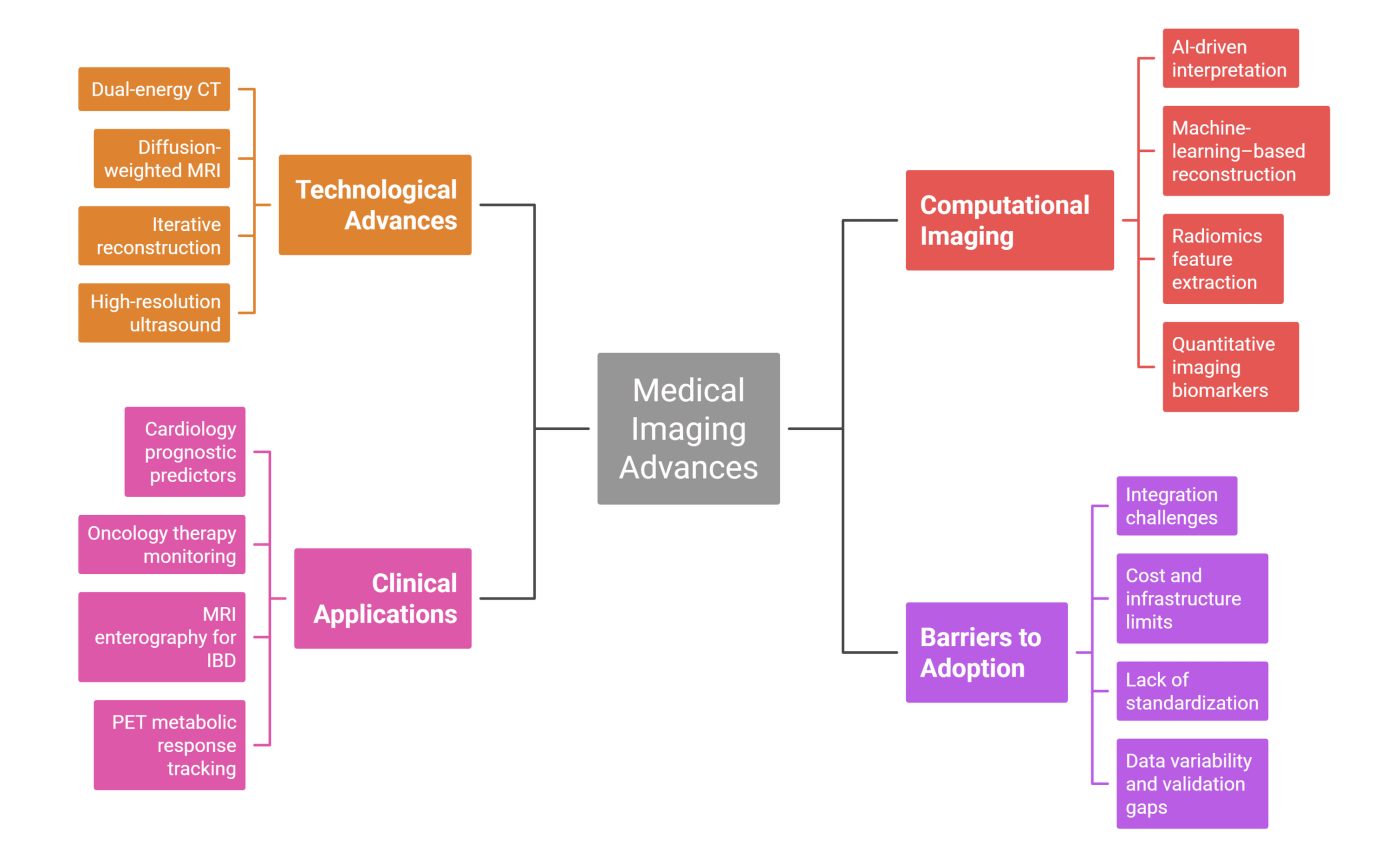
Major domains of medical imaging advances.

Adoption challenges often mirror these advances. High equipment costs and infrastructure requirements continue to restrict access, especially in low- and middle-income countries where advanced modalities remain limited to tertiary hospitals [19]. Global analyses demonstrate significant inequities in imaging access, particularly MRI and PET availability worldwide [15]. Even high-income systems face challenges such as cost-effectiveness, overdiagnosis, and radiation exposure. A further limitation is the lack of longitudinal data supporting imaging biomarker performance in non-oncological diseases [20]. Given these considerations, this narrative review synthesises advances across CT, MRI, PET, ultrasound, and computational imaging, focusing on diagnosis, monitoring, and prognosis in internal medicine. Figure 1 illustrates the interconnected components that define modern imaging developments.

Objectives of the review

This review is guided by four main objectives. First, it seeks to critically evaluate recent advances in radiological imaging and their role in internal medicine, with particular emphasis on the early detection of high-burden conditions such as neurological disorders, cardiovascular disease, and cancer. Second, it examines the contribution of imaging to prognostic assessment and treatment monitoring, with a focus on biomarkers and functional measures. Third, it explores the integration of radiomics and AI into clinical workflows, highlighting their potential to improve diagnostic accuracy as well as the challenges that hinder their adoption. Fourth, it summarizes current limitations related to access, cost-effectiveness, and variability in imaging standards and identifies areas in which evidence remains limited, including the need for stronger validation and improved equity in imaging availability. These objectives provide a structured foundation for understanding both the opportunities and constraints shaping modern radiological practice.

Methodological considerations

In this review, a structured narrative approach was used to maintain transparency and consistency. The literature search was conducted across PubMed, Scopus, Web of Science, and Embase to identify studies published between 2015 and 2025. Radiological imaging, technological advancement, internal medicine disorder, and clinical outcome keywords and controlled vocabulary terms were used. Peer-reviewed, English-language articles were included, as translation services were not available. Eligible studies comprised randomized controlled trials, observational studies, systematic reviews, meta-analyses, and high-impact narrative reviews that examined the diagnostic, monitoring, or prognostic role of imaging. Titles and abstracts were independently screened by two reviewers, and discrepancies were resolved in consultation with a third reviewer. The search identified 612 records. After removal of duplicates, 478 titles and abstracts were screened. A total of 94 full-text articles were assessed for eligibility, and 52 studies met the inclusion criteria and were incorporated into the qualitative synthesis.

As this is a narrative review, no quantitative synthesis, statistical pooling, or meta-analysis was performed, and all findings are presented descriptively. Formal systematic review tools were not applied. However, general study quality and potential risk of bias were considered by assessing the clarity of each study’s methodology, the suitability of the study design for the research question, the completeness of reported outcomes, and the relevance of the findings to the objectives of this review. Qualitative synthesis was conducted by comparing the thematic findings across studies, considering methodological robustness and consistency of reported results, without weighting or statistical aggregation. A standardized template supported consistent data extraction. Because no statistical modeling or pooled effect estimation was undertaken, additional statistical peer review was not required. This approach ensured that included studies were methodologically reliable and aligned with the focus of this review, while remaining appropriate for a narrative synthesis rather than a systematic evaluation.

## Review

Evolution of radiological imaging in internal medicine

Radiological imaging has undergone major transformations that have reshaped diagnostic and therapeutic approaches in internal medicine [17]. The discovery of X-rays in 1895 by Wilhelm Roentgen marked the beginning of non-invasive medical imaging and rapidly expanded from fracture detection to the identification of pulmonary diseases such as tuberculosis and pneumonia, conditions that were widespread during the early twentieth century [18,19]. The availability of early X-ray technology reduced diagnostic delays and contributed to improved survival in affected populations [20]. Recent reviews confirm that early radiographic innovation laid the groundwork for modern imaging development [21].

The development of CT in the 1970s introduced cross-sectional imaging with far greater detail than conventional radiography [21]. CT rapidly became essential for evaluating neurological and cardiovascular conditions, offering clinicians the ability to diagnose stroke, brain tumors, and vascular disease with enhanced accuracy [22]. Its value in trauma assessment, emergency medicine, and pre-surgical planning soon followed, as CT enabled rapid visualization of bone and soft tissue injuries at the point of care [23]. Contemporary analyses highlight CT’s central role in precision diagnostics across internal medicine [22].

MRI, introduced in the 1980s and 1990s, provided detailed soft-tissue visualization without ionizing radiation [24]. MRI proved particularly valuable for central nervous system evaluation, including multiple sclerosis and brain tumor diagnosis [25]. Subsequent advances, such as diffusion tensor imaging and functional MRI, expanded MRI applications to include assessment of neurological connectivity, neural activity, and the effects of injury or disease [26]. The emergence of cardiac MRI in the early 2000s offered precise evaluation of myocardial function and vascularity, improving the diagnostic accuracy of heart failure and ischemic heart disease and supporting more individualized management strategies [27]. Recent literature further emphasizes the expanding role of MRI in personalized medicine and complex disease evaluation [23].

PET, especially in its hybrid form as PET/CT, has become indispensable in oncology by enabling visualization of metabolic activity alongside anatomical structures [28]. This integration has improved the accuracy of staging, treatment planning, and monitoring, contributing to better survival outcomes across multiple cancer types [17]. The subsequent development of PET/MRI further enhanced diagnostic capability by combining high-resolution soft-tissue contrast with metabolic imaging, while avoiding the radiation exposure associated with CT. PET/MRI is particularly valuable for patients requiring repeated imaging in long-term disease monitoring [29]. Hybrid imaging advancements, including PET/MRI, are increasingly recognized as pivotal in modern clinical imaging workflows [30].

**Figure 2:**
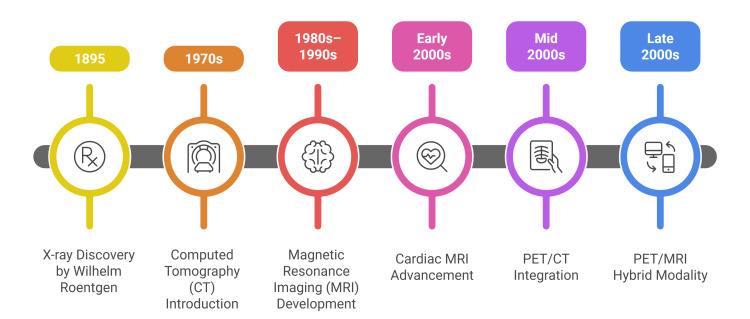
Chronological evolution of radiological imaging modalities in internal medicine.

Together, these technologies demonstrate the progression of imaging from a basic diagnostic tool to an essential system for understanding disease mechanisms, guiding therapies, and tracking patterns of progression [31]. The historical trajectory of imaging in internal medicine, from early X-rays to modern hybrid PET/MRI systems, reflects continuous innovation and expanding clinical utility. Recent comprehensive reviews support this evolutionary framework, underscoring rapid advances in modality integration and quantitative imaging [32]. Figure 2 presents this chronological evolution in a streamlined, modular format that highlights defining advancements across technological eras. Ongoing developments continue to strengthen the role of imaging across specialties and accelerate the adoption of precision medicine strategies.

Role of CT in internal medicine disorders

CT remains one of the most adaptable and widely used imaging modalities in internal medicine [28]. Low-dose CT has been one of its major contributions, allowing substantial reductions in radiation exposure without compromising diagnostic quality [29]. Its value is particularly evident in lung cancer screening, where the detection of small early-stage tumors significantly improves survival [30]. In high-risk individuals, including long-term smokers, low-dose CT has shown far greater sensitivity than standard chest radiography and has contributed to meaningful reductions in lung cancer-related mortality [31].

Recent innovations have expanded CT capabilities. Dual-energy CT improves tissue characterization by acquiring information at two different energy levels, enabling more accurate identification of coronary plaque composition, vascular ischemia, and perfusion deficits [32]. Spectral CT has similarly improved tissue differentiation and is especially useful for diagnosing renal pathology, including kidney stones and renal mass evaluation that may be difficult to assess with conventional techniques [33].

In pulmonary medicine, CT pulmonary angiography is considered the reference standard for diagnosing pulmonary embolism because it provides rapid and precise visualization of thrombi within the pulmonary arteries [34]. This level of accuracy is critical in emergency settings in which timely intervention improves outcomes [35]. In oncology, contrast-enhanced CT supports the detection and staging of cancers of the liver, kidney, and pancreas, and plays a central role in pre-surgical planning, image-guided biopsy, and assessment of treatment response [36]. CT also contributes significantly to treatment monitoring, especially in solid tumors where changes in lesion size guide therapy modification [37]. Because of its speed, broad availability, and consistently high spatial resolution, CT continues to meet the clinical demands of internal medicine and contributes significantly to early diagnosis and improved patient outcomes across a wide range of specialties [38].

MRI innovations

MRI continues to evolve and has become an indispensable modality across neurology, cardiology, gastroenterology, and oncology [39]. Functional MRI has advanced neurological imaging by enabling visualization of brain activity and identifying functional alterations that precede structural changes [40]. These capabilities support earlier diagnosis and monitoring of neurodegenerative diseases, including Alzheimer’s disease and Parkinson’s disease [41]. Cardiac MRI is considered the gold standard for evaluating heart failure, cardiomyopathies, and myocardial infarction because it offers a highly detailed assessment of cardiac structure, scarring, perfusion, and myocardial fibrosis [42]. This level of precision improves clinical decision-making, particularly regarding revascularization strategies, device therapy, and eligibility for heart transplantation [43].

In gastroenterology, MRI enterography has become essential for diagnosing and monitoring inflammatory bowel disease [44]. Its ability to visualize intestinal inflammation, detect complications, and monitor therapeutic response without radiation exposure makes it an invaluable tool for long-term disease management [45]. Magnetic resonance spectroscopy (MRS) further expands MRI capabilities by providing metabolic information about tissues [46]. In oncology, MRS can differentiate benign from malignant lesions and, in selected cases, may serve as a non-invasive alternative to biopsy [47]. It also contributes to research into brain metabolism in epilepsy and neurodegenerative disorders, offering deeper insights into disease progression [48]. MRI has, therefore, become a key component of internal medicine imaging, providing detailed, non-invasive visualization across multiple organ systems and supporting diagnosis, monitoring, and individualized treatment planning [49] (Table 1).

**Table 1:** Clinical applications and innovations of magnetic resonance imaging (MRI) in internal medicine.

Domain	MRI technique	Applications	Clinical impact	Selected references
Neurology	Functional MRI	Maps brain activity and connectivity; monitors Alzheimer’s and Parkinson’s disease	Enables early diagnosis before structural changes; improves disease management and slows progression	[8]
Cardiology	Cardiac MRI	Assesses myocardial infarction, cardiomyopathies, heart failure, scarring, fibrosis, and perfusion	Gold standard for structural and functional evaluation; guides revascularization and transplant decisions	[31]
Gastroenterology	MRI enterography	Evaluates Crohn’s disease and ulcerative colitis (inflammatory bowel disease); monitors therapy response	Provides high-resolution intestinal imaging without radiation; reduces invasive procedures	[15]
Oncology	Magnetic resonance spectroscopy	Differentiates benign vs. malignant lesions; evaluates brain metabolism	Offers metabolic profiling; potential non-invasive alternative to biopsy; deeper insights into tumor biology	[22]
General Medicine	High-resolution structural MRI	Broad-spectrum use for early identification and monitoring of internal medicine disorders	Provides non-invasive, radiation-free imaging; enhances personalized treatment planning	[7]

Nuclear medicine and hybrid imaging (PET/CT and PET/MRI)

Nuclear medicine techniques, particularly PET, have transformed the evaluation of oncology, metabolic disease, and inflammatory conditions [39]. PET uses positron-emitting radiotracers to visualize cellular metabolic activity, allowing clinicians to detect disease at a functional level [40]. In oncology, PET has become central to diagnosis, staging, and treatment response assessment because it identifies primary tumors and metastatic spread with high sensitivity [22]. Hybrid PET/CT combines metabolic information from PET with the anatomical detail of CT, improving diagnostic accuracy and enabling early detection of metastatic disease [41]. PET/CT is especially valuable in non-small-cell lung cancer, where early identification of occult metastases supports more appropriate treatment decisions and improves survival outcomes [10]. PET/CT is also widely used across internal medicine to refine treatment planning and evaluate therapeutic response in multiple malignancies [17].

PET/MRI extends these advantages by integrating PET metabolic data with MRI’s high soft-tissue contrast, offering superior visualization of brain tumors and treatment effects while minimizing radiation exposure [19]. PET/MRI is particularly beneficial in pediatric and neuro-oncologic imaging because it avoids cumulative radiation risk while improving diagnostic precision [25]. The increasing availability of radiotracers such as 18F-fluorodeoxyglucose (FDG) has strengthened PET’s role in detecting early metabolic changes, including those associated with neurodegenerative conditions such as Alzheimer’s and Parkinson’s disease [27]. As hybrid technologies advance, PET-based imaging continues to enhance diagnostic accuracy and early detection across a broad spectrum of internal medicine disorders [28].

Ultrasound and elastography in internal medicine

Ultrasound remains one of the most versatile imaging tools in internal medicine because it is non-invasive, cost-effective, portable, and capable of providing real-time diagnostic information [16]. Point-of-care ultrasound has improved emergency and critical care practice by enabling rapid bedside assessment of cardiac dysfunction, abdominal pathology, and trauma-related injuries [29]. Its ability to guide immediate clinical decisions can reduce diagnostic delays and improve outcomes in emergencies such as hemoperitoneum, where early detection facilitates timely surgical intervention and reduces mortality [30]. Contrast-enhanced ultrasound (CEUS) enhances visualization of vascular patterns within soft tissues and is highly valuable for characterizing liver lesions and differentiating benign from malignant tumors [20]. CEUS has also reduced reliance on invasive biopsy in hepatology and oncology by improving real-time lesion characterization [31].

Elastography measures tissue stiffness and has become foundational in managing chronic liver diseases such as hepatitis C and non-alcoholic fatty liver disease (NAFLD) [45]. Standardized liver elastography enables clinicians to monitor fibrosis progression and determine when antiviral or disease-modifying therapy is required [46]. Thyroid elastography is increasingly used to evaluate the malignancy risk of thyroid nodules, helping avoid unnecessary biopsies [47]. Together, ultrasound and elastography offer radiation-free, accessible imaging options with wide clinical relevance across internal medicine [24].

AI and machine learning in imaging

AI is rapidly transforming diagnostic imaging by enhancing interpretation accuracy, improving workflow efficiency, and reducing variability in routine clinical tasks [29]. Machine learning algorithms assist radiologists by identifying patterns and abnormalities on CT, MRI, and X-ray images with performance comparable to that of experienced specialists [31]. Automated detection of lung nodules on CT and AI-assisted mammography systems that reduce false-negative rates demonstrates the clinical value of AI-driven tools [48]. Radiomics, which extracts quantitative features such as texture, shape, and intensity from standard medical images, provides predictive and prognostic information that complements visual interpretation [49]. Radiomics has shown strong predictive value for recurrence and survival in cancers such as lung cancer and glioblastoma, enhancing treatment response prediction when combined with conventional imaging [50].

AI applications are increasingly integrated into cardiac imaging, including automated segmentation of cardiac MRI to assess myocardial viability, fibrosis, and perfusion [51]. These tools improve measurement consistency, shorten reporting times, and support more personalized management of heart failure and cardiomyopathy [52]. Despite these advantages, AI implementation faces key challenges, including dependence on large and diverse datasets, difficulties in generalizing algorithms across institutions, and limitations related to interpretability and medico-legal responsibility [53]. Additional barriers include limited interoperability with clinical systems, insufficient multicenter validation, and workflow integration challenges in real-world settings [34]. Even with these constraints, AI-supported imaging has demonstrated strong potential to improve diagnostic accuracy, reduce clinician workload, and enhance patient outcomes [35]. Table 2 summarizes the major advantages and limitations of AI applications in cancer detection, cardiac analysis, and general imaging practice.

**Table 2:** Applications of artificial intelligence (AI) and radiomics in diagnostic imaging.

Domain	Application	Clinical impact	Selected references
Oncology (CT, mammography, radiomics)	AI detects lung nodules on CT; classifies benign vs. malignant; reduces mammogram false negatives by 20%; radiomics predicts recurrence and survival in lung cancer, glioblastoma, and other solid tumors	Improves early cancer detection and survival; enhances predictive accuracy of treatment response by up to 30%; reduces unnecessary biopsies	[29]
Breast imaging	AI applied to mammography for automated lesion detection	Reduces false negatives; increases the accuracy of breast cancer screening; improves survival through early detection	[5]
Cardiology (cardiac MRI + AI)	Automatic segmentation of heart structures; evaluation of myocardial perfusion, fibrosis, and viability	Faster, consistent cardiac analysis; saves time in workflow; enables more personalized treatment planning	[18]
General imaging (CT, MRI, X-ray)	AI algorithms detect patterns and abnormalities with specialist-level accuracy	Automates routine interpretation; reduces clinician workload; improves reproducibility and efficiency	[47]
Limitations	Lack of large annotated datasets, poor interoperability with clinical systems, and limited regulatory acceptance	Restricts large-scale adoption; requires multicenter validation and international standards for clinical integration	[38]

Radiomics and imaging biomarkers

Radiomics is an emerging field that extracts quantitative image features such as texture, shape, and intensity to support diagnosis, prognosis, and treatment planning [54]. These features capture subtle patterns within tissues that are not visible to the human eye, providing insights into disease heterogeneity and biological behavior [28]. Radiomics has shown particular promise in oncology, where it can assess tumor heterogeneity, monitor therapeutic response, and help identify early biomarkers of disease [55]. In non-small-cell lung cancer, specific radiomic signatures have been linked to survival outcomes and treatment response, and CT-based radiomic models have predicted tumor recurrence with reported accuracies as high as 86% [56].

Radiomics has also been applied to mammography, where three-dimensional radiomic features have been used to characterize tumor aggressiveness and predict lymph node involvement, improving staging accuracy and treatment planning [57]. Beyond oncology, radiomics contributes to cardiovascular and liver disease assessment. Cardiac MRI radiomics can quantify myocardial tissue characteristics such as fibrosis or edema, supporting early prediction of heart failure progression, while radiomics-inspired elastography techniques help predict liver fibrosis development in chronic hepatitis and NAFLD [58].

Despite these benefits, radiomics faces challenges related to variability in imaging protocols, scanner differences, and a lack of standardization across institutions [17]. Reliable clinical adoption requires validation of radiomic biomarkers in large, multicenter cohorts to ensure reproducibility and generalizability [59]. As machine learning algorithms continue to advance, radiomics is expected to play an increasingly important role in precision medicine by supporting individualized therapy selection and improving early disease detection [29].

Imaging in the early diagnosis of internal medicine disorders

Imaging plays a central role in the early detection of internal medicine disorders by identifying disease before significant clinical deterioration occurs [22]. Early detection is especially important in conditions where timely intervention reduces morbidity and mortality, including cancer, cardiovascular disease, and neurodegenerative disorders [14]. Low-dose CT is now the standard screening modality for lung cancer in high-risk individuals, such as smokers, and has been shown to reduce lung cancer mortality by 20% by detecting small, early-stage tumors that are not visible on standard chest radiography [9,35].

Similarly, three-dimensional mammography has improved breast cancer screening accuracy by lowering false-positive rates and enabling earlier detection, which increases the likelihood of successful treatment [41]. In cardiovascular medicine, CT coronary angiography (CTA) is now routinely used to evaluate coronary artery disease (CAD). CTA improves diagnostic accuracy for CAD by approximately 25% and helps clinicians determine whether interventions such as angioplasty or bypass surgery are needed [27,52].

**Figure 3:**
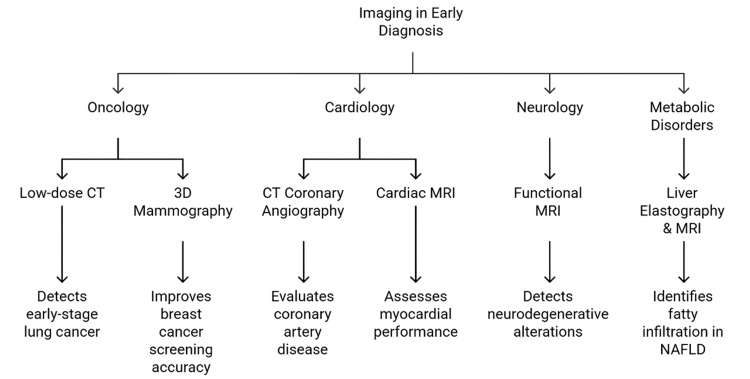
Applications of imaging modalities in the early diagnosis of internal medicine disorders.

MRI also contributes substantially to early detection. Cardiac MRI provides high-resolution assessment of myocardial function and scar tissue, supporting early diagnosis of heart failure and ischemic heart disease [13]. Neurodegenerative diseases such as Alzheimer’s and Parkinson’s disease often present subtly in their earliest stages [43]. Functional MRI can identify early changes in brain connectivity and neural activity, offering the potential for earlier diagnosis and intervention [11,37]. Imaging is equally important for the early detection of metabolic disorders. Liver elastography and MRI can identify early fatty infiltration and fibrosis in NAFLD, enabling timely lifestyle or therapeutic interventions to prevent progression to cirrhosis or hepatocellular carcinoma [4,25]. Early diagnosis through imaging not only improves clinical outcomes but also reduces long-term healthcare costs by avoiding late-stage disease requiring more intensive treatments [19]. Modalities used for early diagnosis across major internal medicine conditions are summarized in Figure 3.

Disease monitoring and prognostic assessment imaging

Along with early diagnosis, imaging plays a central role in monitoring disease progression and informing prognostic assessment across internal medicine specialties [27]. In chronic disorders such as cancer, cardiovascular disease, and rheumatoid arthritis, serial imaging allows clinicians to evaluate disease activity, assess treatment response, and adjust therapeutic strategies to improve long-term outcomes [18]. In oncology, PET/CT is widely used to monitor patients undergoing chemotherapy or immunotherapy [34]. PET evaluates tumor metabolism, and reductions in FDG uptake during treatment serve as early markers of therapeutic response, enabling clinicians to modify ineffective regimens and improve survival outcomes [12]. This metabolic imaging approach supports earlier adjustment of therapy when treatment response is inadequate [25]. PET-based monitoring has been associated with improved survival by helping clinicians identify non-responders sooner [36].

In cardiology, cardiac MRI is routinely used to assess myocardial scarring and tissue viability in heart failure and ischemic heart disease [14]. MRI can detect subtle abnormalities in myocardial function and early patterns of scarring, which support risk stratification and guide interventions aimed at preventing arrhythmias or disease progression [29]. Additional MRI-derived markers, such as late gadolinium enhancement, provide prognostic insights that help anticipate clinical deterioration [31]. In rheumatology, ultrasound and MRI have become essential for evaluating joint inflammation and structural damage in conditions such as rheumatoid arthritis [15]. MRI is particularly useful for detecting early synovitis before changes appear on X-ray studies, allowing clinicians to initiate disease-modifying therapy that prevents long-term joint destruction [23]. Ultrasound also supports real-time monitoring and image-guided biopsy for ongoing disease assessment [37].

Across specialties, imaging contributes significantly to prognostic modeling. PET/CT helps estimate recurrence risk in oncology [13], while cardiac MRI supports the prediction of adverse cardiac events and guides long-term management of patients with heart failure or arrhythmias [19]. These imaging-derived prognostic metrics enable clinicians to individualize follow-up schedules, optimize therapy, and reduce long-term complications [32]. Thus, imaging remains an indispensable component of comprehensive prognostic assessment in internal medicine.

Challenges in clinical translation and cost-effectiveness

Despite major technological advances, several challenges continue to limit the widespread clinical adoption of advanced imaging modalities. High cost, variable access, and inconsistent insurance coverage create substantial barriers, particularly in resource-limited regions [20]. PET/CT and MRI are expensive to install, maintain, and operate, and even many tertiary hospitals do not have ready access to these systems [35]. This leads to marked disparities in care between urban centers and rural or low-income settings [16]. Cost remains one of the most significant obstacles to broader implementation. PET/CT scanners require multimillion-dollar investments and significant operational expenses, and many insurance providers restrict coverage for certain indications or patient groups [10,21]. As a result, health systems place increasing emphasis on evaluating the cost-effectiveness of advanced imaging technologies, especially in low-income environments [28].

Another concern is the possibility of imaging overuse. Large-scale screening programs, such as low-dose CT for lung cancer, may detect small pulmonary nodules that have little clinical relevance, leading to additional imaging, invasive procedures, increased healthcare costs, and unnecessary patient anxiety [26,33,38]. AI and machine learning hold promise for overcoming some of these limitations. AI-driven tools can automate image interpretation, improve diagnostic precision, and streamline workflows, potentially reducing overall costs [17]. However, significant barriers remain: AI systems require large, diverse datasets; concerns persist about transparency, bias, and data privacy; and regulatory pathways for clinical approval are not well established [40].

Advanced imaging technologies have enormous potential to improve patient care, but their real-world impact depends on balancing clinical benefit with cost, accessibility, and appropriate utilization [22]. Strengthening affordability, expanding access, and promoting efficient use will be essential for maximizing the benefits of these modalities in diverse healthcare settings worldwide [30]. Table 3 summarizes the major limitations of advanced imaging, including high costs, unequal access, risks of overuse, and challenges associated with AI integration.

**Table 3:** Challenges and considerations in the clinical use of advanced imaging technologies.

Challenge area	Description	Clinical impact	Potential solutions	References
Access and affordability	PET/CT and MRI are expensive to install and operate; not widely available in low-resource or rural hospitals	Creates disparities in care; limits access to advanced diagnostics in many regions	Develop cost-cutting strategies, expand access, and prioritize equitable distribution of technologies	[39]
Cost burden	Imaging procedures may not be covered by insurance; high operational costs strain healthcare budgets	Reduces patient access; limits adoption in low-income countries; raises questions of cost-efficiency for health systems	Policy reforms, inclusion in insurance coverage, and investment in cost-efficient imaging approaches	[8]
Overuse in screening	Low-dose CT lung screening may detect clinically insignificant nodules	Leads to unnecessary follow-up scans, biopsies, treatments, added costs, and patient anxiety	Establish clear guidelines, use artificial intelligence (AI) to refine risk stratification, and ensure appropriate utilization	[27]
AI integration challenges	AI and machine learning can improve accuracy and efficiency, but face issues of high development cost, lack of transparency, data privacy, and regulatory barriers	Slows adoption; raises ethical and legal concerns in clinical deployment	Promote algorithm transparency, strengthen data governance, ensure regulatory clarity, and validate through multicenter studies	[22]
Overall consideration	Balancing innovation with affordability and equitable access	Potential underutilization of transformative imaging technologies	Strategic health policy, global cooperation, and careful evaluation of clinical benefit vs. cost	[14]

Limitations and future recommendations

Despite major progress in imaging technologies, several limitations continue to restrict their widespread clinical use. Differences in imaging protocols, reconstruction parameters, and study designs across institutions lead to variability in diagnostic accuracy and hinder reproducibility. Variations in CT acquisition settings, including slice thickness and contrast timing, can result in subtle lesions being missed or inconsistently detected between centers. Similarly, the absence of standardized imaging biomarkers across platforms limits the ability to compare results, pool data, and validate quantitative tools such as radiomics in routine practice. Cost remains a major barrier to access. Advanced modalities, including PET/CT and MRI, require substantial financial investment for installation, maintenance, and staffing, making them difficult to implement in resource-constrained or rural settings. These inequalities in availability reinforce global disparities in diagnostic capacity and delay early detection for populations already at higher clinical risk.

Looking ahead, integrating AI with radiomics offers major potential to enhance diagnostic accuracy, improve prognostic modeling, and support personalized treatment planning. Hybrid modalities such as PET/MRI provide complementary metabolic and anatomic information that can strengthen early detection and improve treatment planning, particularly in oncology. However, the clinical impact of these innovations depends on achieving consistent data quality, establishing standardized imaging protocols, and conducting large multicenter validation studies to ensure generalizability. Regulatory clarity and high-quality datasets will also be essential for safe and responsible deployment of AI-based tools. Addressing these challenges will be critical for expanding the clinical adoption of advanced imaging and promoting more equitable access to these technologies worldwide.

## Conclusions

This review underscores how advanced imaging technologies are reshaping internal medicine by enhancing early diagnosis, supporting continuous disease monitoring, and refining prognostic assessment across a wide spectrum of conditions. A key insight is the importance of a multi-modality framework in which AI, radiomics, MRI, and PET/CT complement one another to strengthen diagnostic precision and promote more individualized, patient-centered care. These integrated innovations demonstrate the growing capability of technology to provide clinicians with deeper insights into disease mechanisms and support more timely and informed decision-making. The broader adoption of these modalities remains challenged by economic limitations, lack of standardized imaging protocols, and disparities in access between regions. Achieving equitable implementation will depend on collaborative efforts among researchers, clinicians, and policymakers to establish uniform guidelines, improve cost-effectiveness, and ensure that the benefits of these technologies are accessible worldwide. Integrating these tools responsibly can advance precision medicine and improve long-term healthcare outcomes.
